# Allium-sativum and bakuchiol combination: A natural alternative to Chlorhexidine for oral infections?

**DOI:** 10.12669/pjms.36.2.1457

**Published:** 2020

**Authors:** Ayesha Fahim, Wan Harun Himratul-Aznita, Puteri Shafinaz Abdul-Rahman

**Affiliations:** 1 Dr. Ayesha Fahim, M. Phil, Department of Oral & Craniofacial Sciences, Faculty of Dentistry, University of Malaya, Kuala Lumpur, Malaysia; 2 Dr. Wan Harun Himratul-Aznita, PhD. Department of Oral & Craniofacial Sciences, Faculty of Dentistry, University of Malaya, Kuala Lumpur, Malaysia. Faculty of Dental Medicine, Universitas Airlangga, Surabaya, Indonesia; 3 Dr. Puteri Shafinaz Abdul-Rahman, PhD. Department of Molecular Medicine, Faculty of Medicine, University of Malaya, Kuala Lumpur, Malaysia

**Keywords:** *Allium sativum*, *Bakuchiol*, Candida, Chlorhexidine, Streptococci

## Abstract

**Objective:**

Chlorhexidine mouthrinses are considered a gold standard as an adjunct treatment of oral infections. However, owing to its toxicity, discoloration of tooth surface and the emerging prevalence of drug-resistant species, attention is being given to exploring natural alternatives to the drug.

**Methods:**

The experiment was carried out in Azra Naheed Center for Research and Development (ANCRD), Superior University, Lahore, Pakistan from September 2018 till May 2019. Biofilms and planktonic cells of *C. albicans* alone and in combination with streptococci were subjected to chlorhexidine, *allium sativum* and *bakuchiol* individually and to *allium*-*bakuchiol* combination. Kirby-Bauer test, antifungal susceptibility testing, CFU count and drug synergy assessment was done on planktonic cells. Dynamic biofilms were formed to mimic conditions similar to oral cavity and CFU was determined.

**Results:**

MIC of all three agents was higher against mixed species when compared to single species planktonic cells and biofilm. *Allium sativum* and *bakuchiol* demonstrated synergistic effects. The decrease in CFU count and minimum biofilm reduction to salivary pellicle caused by *allium sativum-bakuchiol* was comparable to that of chlorhexidine.

**Conclusion:**

Thus, *allium sativum*-*bakuchiol* combination demonstrated antimicrobial effects similar to chlorhexidine against planktonic cells and dynamic biofilm. It could serve as a possible natural, economical alternative to chlorhexidine mouthrinses usually recommended in dental clinics. However, *in vivo* studies are required to determine the correct dosage of these agents.

## INTRODUCTION

Chlorhexidine mouth rinses (0.2% w/v) are considered a gold standard as an adjunct treatment of oral bacterial and fungal infections in dental clinics.^1^ However, the therapeutic applications of chlorhexidine become limited owing to its toxicity, discoloration of tooth surface and the emerging prevalence of drug-resistant candidal species^1^. Antimicrobial resistance is a consequence of imprudent use of antimicrobial agents and develops when a pathogenic organism mutates or obtains a resistance gene.^2^ *Candida albicans* infections are becoming an escalating challenge for doctors as they are opportunistic microorganisms colonizing the human oral cavity^2^. *Streptococcus mitis* and *Streptococcus sanguinis*, commensals of oral cavity have been co-isolated with *C. albicans* from several infections and from various biomaterial surfaces including implants, dentures, voice prostheses, feeding tubes and catheters.^3^ This type of polymicrobial growth may not only alter the inherent virulence of the species, but the treatments normally effective against monomicrobial species infections may also be rendered futile.^4^ When such infections are not cured by traditional therapy, doctors shift to steroids and harsher measures.

Natural plant derived products can be used because of antimicrobial properties and minimal side effects on human health.^5^ *Bakuchiol*, derived from leaves of *Psoralea glandulosa* (Culen), is commonly used in folk medicine for the treatment of skin diseases caused by bacteria and fungi. *Allium sativum* (garlic) belonging to *Liliaceae* family, also has antibacterial activity against many common pathogenic bacteria.^6^ Preparing drugs in combination is common in modern medicine to enhance the pharmaceutic efficacy and decrease the side effects from the excessive use of drugs.

The aim of present research is to compare the effects of 0.2% w/v chlorhexidine, *A. sativum* extract and *bakuchiol* extract on planktonic and biofilm form of *Candida albicans*. The latter will be tested both alone and in a mixed species biofilm including *Streptococcus mitis* and *Streptococcus sanguinis*, which are early colonizers of oral cavity.

## METHODS

### Inoculum and Media

The experiment was carried out in Azra Naheed Center for Research and Development, Superior University, Lahore from September 2018 till May 2019 with the approval of the Institutional Research Ethics Board (Ref: 40550/AN/SU, dated March 7, 2017). Written informed consent was obtained from the individuals who participated in this study.*C. albicans* (ATCC 14053), *S. sanguinis* (BAA 1455) and *S. mitis* (ATCC 49456) strains were used for the formation of *in vitro* biofilms. A general purpose medium tryptic Soy Broth (Merck) was used as nutrient media.

### The Kirby-Bauer Susceptibility Test

The antimicrobial activity of chlorhexidine (CHX) (Merck), *Bakuchiol* (Bk) (Merck) and *Allium sativum* (As) (Merck) (the last two used both alone and in combination) was analyzed by disc-diffusion susceptibility test proposed by Kirby-Bauer.^7^ Single and mixed species suspension was swabbed uniformly over a Mueller Hinton agar (MHA) plate. Sterile 6mm paper discs (Whatman^®^, USA) saturated with 100 µL of CHX (0.2% w/v), As (50µg/mL) and Bk (50µg/mL) each were placed on the inoculated agar surface. For As + Bk, 50 µL of each was used. A blank paper disc inoculated with sterile distilled water was used as negative control. All plates were incubated at 37°C for 24 h. The inhibition zone created around the disc was measured to estimate the antimicrobial susceptibility for each group. The experiment was repeated in triplicate with biological and technical variants (*n* = 9) and mean value was recorded.

### Antifungal susceptibility test of planktonic cells

Based on the results obtained from Kirby-Bauer test the minimum inhibitory concentration (MICs) of CHX, As and Bk for single and mixed species were determined using the Clinical and Laboratory Standards Institute broth microdilution method (CLSI-BMD) described in NCCLS guidelines.^8^ A 100 µL of single species (candida alone) and mixed (candida with both streptococci) (ratio of 1:1:1 at similar respective concentrations of 1 x 10^6^ cells / mL) species inoculum prepared in TSB was poured in 96-well plates, followed by 100 µL of different concentrations of CHX, As and Bk (125, 100, 64, 50, 32, 16, 8, 4, 2 µg / mL respectively. Tryptic soy broth, free of antimicrobial agent or active compounds was used as positive control and TSB without addition of microorganism was used as negative control. After 24hours’ incubation at 37°C, organism growth was assessed by optical density OD_550nm_ using a spectrophotometer (FC-Bios µQuant).

The MIC was determined as the minimum concentration of antimicrobial agent that inhibited ≥ 50% of microbial growth in comparison to positive control using a modified Gompertz model. The experiment was performed three times to ensure reliable and reproducible results.

### Determination of drug synergy against planktonic cells

Combination of As with Bk was assessed for synergistic affect against planktonic single and mixed species using the checkerboard microdilution method.^9^ Different concentrations of antifungal combination (125, 100, 64, 50, 32, 16, 8, 4, 2 µg / mL) at 100 µL volume were poured in a sterile microtiter plate. Following which, 100µL of single and mixed species *Candida* suspension of 10^6^ cells/mL was added. The plates were then incubated at 37°C overnight and assessed in spectrophotometer). MIC and Fractional inhibitory concentration (FIC) was determined using the MIC values of As + Bk. The sum of individual FICs; FIC index was determined using the following formula:

FICi = MIC(As_comb_)/MIC(As_alone_) + MIC(Bk_comb_)/MIC(Bk_alone_).

FICi ≤ 0.5= synergy; FICi >0.5- ≤1= partial synergy; FICi >1- ≤4= indifference; and >4, antagonism.^9^

### Production of dynamic biofilm

Biofilm was formed using Nordini’s artificial mouth model (NAM) defined by Rahim et al. (2008)^10^ by using glass beads to mimic tooth surface. Continuous salivary flow and 37^o^C temperature was maintained for pellicle formation. After which microorganisms were allowed to grow and form biofilm for 24 h. The biofilm was treated with MIC concentration of CHX, As, Bk and As + Bk combination respectively and CFU was calculated using formula:


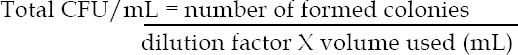


Mean of CFU/mL were calculated in triplicates. The percentage reduction of microbial population in adhesion to salivary pellicle after treatment was calculated as follows:

Percentage decrease in adherence 
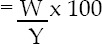


Where “W” is the mean microbial population treated with As + Bk combination and “Y” is the mean microbial population subjected to distilled water (negative control).

### Statistical analysis

All results were calculated and expressed as mean values with standard deviation (±SD) from three technical and biological variants that were performed in triplicate (*n* = 9). SPSS software (version 18.0) was used to perform statistical analyses. Independent t-test was used to distinguish data between single and mixed species group. ANOVA was used to compare differences between groups treated with various drugs. *P* value of less than 0.05 was deliberated as statistically significant.

## RESULTS

### The Kirby-Bauer susceptibility test

All agents created inhibition zones on MH agar. CHX created larger inhibition zone in comparison to As and Bk alone. However, the combination of As and Bk exhibited the largest inhibitory zone in single and mixed species (Table-I) indicating high antimicrobial activity.

**Table-I:** The mean diameter of inhibition zone produced by antimicrobials on single and mixed species.

Groups	Antimicrobial activity (mm) of:				

	Antimicrobials used alone			Combination of agents	

	As (50µg/ml)	Bk (50µg/ml)	CHX (0.2%w/v)	As +Bk (50µg/ml+50µg/mL)	Sterile dH_2_O
Single species (C. albicans alone)	19	20	22	25	NS
Mixed species (Candida and bacteria)	16	18	18	22	NS

NS: no sensitivity.

### Antimicrobial susceptibility of Planktonic cells:

MIC values of CHX, As and Bk was substantially high for mixed species making mixed species susceptible to chlorhexidine only at high dosage. The combination of As and Bk showed synergistic effect against single and mixed species group and was affective against microbes at low dosage (∑FIC index = 0.5) (Table-II).

**Table-II:** Mean antimicrobial susceptibility values of single (Candida albicans) and mixed (Candida-bacteria) species to chlorhexidine, allium sativum, bakuchiol and A. sativum-bakuchiol combination.

Species	Chlorhexidine (MIC) (µg/mL)	Allium sativum (MIC) (µg/mL)	Bakuchiol (MIC) (µg/mL)	As+Bk (MIC) (µg/mL)	FIC index
C. albicans alone	4	8	25	4_A_ + 12.5_B_	0.5= SYN
Mixed species	32	16	50	8_A_ + 12.5_B_	0.5= SYN

***Note:*** The MIC_50_ was determined as the minimum concentration of antimicrobial agent that inhibited ≥ 50% of microbial growth in comparison to drug free control. Fractional inhibitory concentration (FIC) was calculated to determine the synergistic interaction between A. sativum and bakuchiol, *SYN: Synergistic

### Effect of Allium sativum + Bakuchiol on Biofilm

**Fig.1:**
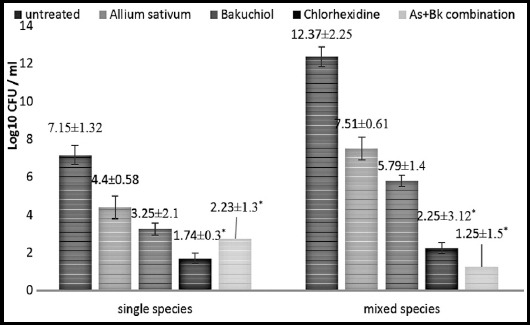
CFU count of single (candida alone) and mixed (candida and bacteria) species after exposure to As, Bk, chlorhexidine and As+Bk. (*) indicates the significant difference between untreated and treated samples (P < 0.05). Given values are represented as mean±S.D.

There was higher microbial count in mixed species in comparison to single species. CHX was extremely effective against single species. However, for mixed species, both As+Bk and CHX were equally effective against microorganisms (p>0.05) (Fig.1).

## DISCUSSION

*C. albicans* is known to form synergistic interactions with a number of bacteria, collectively causing diseases like oral candidiasis, denture stomatitis, periodontitis which are resistant to conventional antifungal treatment.^11^ The increased resistance to commonly prescribed antifungals motivate search for new ways of combating polymicrobial biofilms.

According to previous studies, CSH of planktonic microbial cells positively correlate with formation of biofilm.^12^ Difference in cell surface physicochemical properties and presence of carbohydrate moiety may impact microbial cell coherence to hydrophobic interface. The metabolites and crude extracts derived from herbs and plants are important in the search for new antifungal agents.^13^ *Bakuchiol* has been widely used for skincare regimens, as anti-wrinkle and anti-aging agent^13^ and has previously been linked with antimicrobial properties.^14^ It has been reported that garlic extract (*Allium sativum*) can inhibit growth of both Gram-positive and Gram-negative bacteria. The garlic cloves consist of sulfur containing chemicals like allicin, alliin, and ajoene.^15^ When the garlic cloves are cut or crushed they release the enzyme alliinase which converts alliin to allicin which is responsible for antibacterial activity.^16^ The antibacterial property of garlic extract was evaluated in previous studies and can inhibit the bacterial growth when used at higher concentration.^17^ Similarly, *Bakuchiol* has been studied for its action against C*andida albicans* and non albicans species.^5^ Katsura et al., 2001^14^ has studied the anti-adherence effect of *bakuchiol* on various oral bacteria. The mechanism of action of this compound is yet to be studied in detail. But neither of these agents have been studied against fungal-bacterial mixed species. In the current research, exposure of Candida and mixed species planktonic cells to As+Bk revealed significant decrease in the CSH percentage in comparison to untreated samples. The current research was carried out to assess the antimicrobial activity of *Allium sativum* and *Bakuchiol* combination against candida-bacteria mixed species planktonic and biofilm cells. The results were compared with *Allium sativum*, *Bakuchiol* and chlorhexidine used alone

It was also noted that CHX required a greater MIC against mixed species in comparison to single species. This indicates a stronger interaction between cells when Candida and bacteria are grown together.^18^ This interkingdom bond requires larger dose to overcome microbial growth. The FIC index of As+Bk showed synergy in planktonic cells. The effect of agents was further analyzed by assessing their action against single and mixed species biofilms. Since microorganisms form salivary biofilms in oral cavity and the complexity that is orchestrated in those biofilms govern their virulence and pathogenicity in the oral soft and hard tissue.^5^ The results indicate that As+Bk can be as effective as chlorhexidine against mixed species biofilms.

This study revealed that *Allium sativum* and *Bakuchiol* exposure had a considerable effect towards polymicrobial interactions of Candida-bacterial cells. *Allium sativum* and *Bakuchiol* could have altered cell surface area and caused impairment of cell hydrophobicity which might have led to reduced adhesion to hydrophobic surface.^19^ Clinical trials are needed for confirmation of the *in-vitro* beneficial antimicrobial effect and any needed dosage adjustment, these herbal and locally available agents could be effectively incorporated in the formulation of mouthwashes, gels or / and lozanges for the treatment of oral candidal and adjunctive infections.

## CONCLUSION

Within the limitations of this study, the following could be concluded: *Allium sativum* and *Bakuchiol* individually, had antifungal and antimicrobial properties against *C. albicans* alone and candida-bacteria mixed species. The combined effect of *A. sativum* and *bakuchiol* extracts in terms of antimicrobial effects was comparable to that of the chlorhexidine, thus indicating their possible future employment as an alternative to chlorhexidine itself.

### Authors’ Contribution:

**AF** did data collection, manuscript writing & statistical analysis.

**WHHA** was responsible for data accuracy and integrity of the work.

**PSAR** edited and approved the manuscript.
